# Prognostic Significance of Galectin-1 but Not Galectin-3 in Patients With Lung Adenocarcinoma After Radiation Therapy

**DOI:** 10.3389/fonc.2022.834749

**Published:** 2022-02-23

**Authors:** Chun-Chieh Huang, I-Chieh Chuang, Yu-Li Su, Hao-Lun Luo, Ya-Chun Chang, Jo-Ying Chen, Chang-Chun Hsiao, Eng-Yen Huang

**Affiliations:** ^1^ Department of Radiation Oncology, Kaohsiung Chang Gung Memorial Hospital and Chang Gung University College of Medicine, Kaohsiung, Taiwan; ^2^ Graduate Institute of Clinical Medical Sciences, College of Medicine, Chang Gung University, Taoyuan, Taiwan; ^3^ Department of Anatomical Pathology, Kaohsiung Chang Gung Memorial Hospital and Chang Gung University College of Medicine, Kaohsiung, Taiwan; ^4^ Division of Hematology-Oncology, Department of Internal Medicine, Kaohsiung Chang Gung Memorial Hospital and Chang Gung University College of Medicine, Kaohsiung, Taiwan; ^5^ Department of Urology, Kaohsiung Chang Gung Memorial Hospital and Chang Gung University College of Medicine, Kaohsiung, Taiwan; ^6^ Department of Internal Medicine, Kaohsiung Municipal Min-Sheng Hospital, Kaohsiung, Taiwan; ^7^ Division of Pulmonary and Critical Care Medicine, Kaohsiung Chang Gung Memorial Hospital and Chang Gung University College of Medicine, Kaohsiung, Taiwan; ^8^ School of Traditional Chinese Medicine, Chang Gung University College of Medicine, Taoyuan, Taiwan

**Keywords:** galectin-1, galectin-3, lung adenocarcinoma, radiation therapy, radiation response biomarkers

## Abstract

**Introduction:**

To investigate the role of tumor galectin-1 and galectin-3 in patients with lung adenocarcinoma after definitive radiation therapy.

**Methods:**

A total of 41 patients with localized lung adenocarcinoma undergoing thoracic radiation therapy without concurrent chemotherapy were enrolled. Their paraffin-embedded lung tissues were sent for immunohistochemical staining for galectin-1 and galectin-3. The clinical treatment outcomes, including overall (OS), locoregional progression-free (LRPFS), and distant metastasis-free (DMFS) survivals, were evaluated. Univariable and multivariable Cox regression analyses were applied.

**Results:**

Overexpression of tumor galectin-1 and galectin-3 were found in 26.8% and 19.5% of patients, respectively. Overexpression of tumor galectin-1 was the most significant prognosticator to predict worse LRPFS in both univariable (*p* = 0.007) and multivariable analyses (*p* = 0.022). Besides, patients with overexpression of tumor galectin-1 had a trend of worse OS (*p* = 0.066) than those with low expression in multivariable analysis, and worse DMFS (*p* = 0.035) in univariable analysis. The overexpression of tumor galectin-3 had no significant effect on survival outcomes.

**Conclusions:**

The overexpression of tumor galectin-1, but not galectin-3, is associated with poor LRPFS of patients with lung adenocarcinoma after thoracic radiation therapy. Future research on the mechanism of galectin-1 affecting radiation response in lung adenocarcinoma may be worth exploring.

## Introduction

Lung cancer is one of the leading cancers with high incidence and mortality rates in the world. In Taiwan, lung adenocarcinoma is the most common histological type of lung cancer. According to the Taiwan Cancer Registry Annual Report 2018 (1), lung adenocarcinoma accounted for 58% in men and 88% in women with a new diagnosis of lung cancer. The clinical managements to treat lung adenocarcinoma to get better tumor control and survival outcomes are of great value.

Radiation therapy is an important local treatment modality for lung adenocarcinoma (2, 3). Short course of stereotactic ablative radiation therapy is a standard local therapy for medically inoperable early-stage lung adenocarcinoma (4). Long course of definitive radiation therapy plays a major role in the locoregional treatment of locally advanced lung adenocarcinoma (5). For metastatic lung adenocarcinoma, local radiation therapy is recommended for palliation of symptoms and can be used for consolidation in oligo-metastatic or oligo-residual tumors (6). For brain metastasis, both radiosurgery and whole brain irradiation are important treatment options to control intracranial tumors (7). Therefore, the method to improve radiation response and the study on the radioresistance in lung adenocarcinoma are necessary.

Galectins are a family of proteins with binding specificity for beta-galactoside sugars by glycosylation and fifteen mammalian galectins have been identified till now. Galectin-1 is a homodimeric protein encoded by the LGALS1 gene, and galectin-3 is the only chimeric lectin of the galectin family encoded by the LGALS3 gene. Both galectin-1 (8–10) and galectin-3 (11–13) have been found to be involved in tumor development and progression, such as cell proliferation, invasion, and metastasis in lung cancer. As for radiation response, our previous studies first found that galectin-1 was associated with radioresistance in the cervical cancer cell line (14) and was a poor prognostic factor in the patients with squamous cell carcinoma of uterine cervix (15) after definitive radiation therapy. We also proposed that overexpression of galectin-1 was an independent prognosticator in the patients with glioblastoma multiforme (16) after adjuvant radiation therapy, and in the patients with locally advanced esophageal cancer (17) after preoperative chemoradiotherapy. Consequently, in this study, we investigated the prognostic impact of galectin-1 overexpression as well as galectin-3 overexpression on the patients with lung adenocarcinoma after definitive radiation therapy.

## Materials and Methods

### Patients

This study was approved by the Chang Gung Medical Foundation Institutional Review Board (IRB) (No. 201800074B0). The need for individual informed consent from each patient was waived by the IRB as minimal risk to the patients who were already diagnosed and treated. This study was initiated in February 2018. A total of 466 patients diagnosed with lung cancer undergoing thoracic radiation therapy at least 50 Gy in our institution without concurrent chemotherapy between 1987 and 2002 were recorded in our radiation therapy registry database. Patients were staged by thoracic computed tomography including lower neck and upper abdomen area, bone scintigraphy, and brain magnetic resonance imaging.

The following patients were sequentially excluded: 138 with previous thoracic operation, 49 with cytology only or without registry of histopathology type, 226 with non-adenocarcinoma, and 12 with distant metastasis before radiation therapy or inadequate paraffin-embedded tissues for immunohistochemical study. As a result, the pathological tissues of lung adenocarcinoma from 41 patients were enrolled for galectin-1 and galectin-3 immunostainings in this study. Other clinical data including gender, date of birth, initial diagnosis date, first visit date in our department, cancer stage, radiation therapy, neoadjuvant chemotherapy, treatment response, and follow-up status were also acquired for analysis.

### Radiation Therapy

Patients were treated by conventional (27 patients) or three-dimensional conformal (14 patients) radiation therapy. The gross tumor volume comprised primary lung tumor and enlarged regional mediastinal lymph nodes with a short diameter of 1 cm at least. The clinical target volume was gross tumor volume plus 0.5 cm margin to cover microscopic extension. The margin around clinical tumor volume to develop planning target volume was 0.5–1.0 cm to cover setup uncertainty and respiratory motion according to lung tumor location. Two to three radiation portals with Cerrobend blocks or multileaf collimators were applied to cover the planning target volume. Most patients were prescribed with a radiation dose of 60–66 Gy in 1.8–2.0 Gy per fraction. After the completion of radiation therapy, the following are carried out: follow-up of chest x-ray and computed tomography within 1–3 months to evaluate treatment response, and then chest x-ray every 1–3 months, computed tomography every 3–6 months, bone scintigraphy and brain magnetic resonance imaging annually, or when clinically indicated.

### Immunohistochemistry

The detailed process of tissue microarray construction and immunohistochemistry has been described as in our previous studies (15, 16). Briefly, areas presenting histopathologic characteristics of lung adenocarcinoma were selected on the H&E slides. The representative areas were marked on the corresponding paraffin block. The tissue cylinders were taken from the selected areas of the donor paraffin block. Then, they were punched into the recipient paraffin block by tissue-arraying instruments. Several 1-μm-thick sections were cut and mounted on the slides. They were stained with antibodies for galectin-1 (HPA000646, rabbit to human; Sigma-Aldrich, St. Louis, MO) and for galectin-3 (Santa Cruz B2C10).

**Figure 1:**
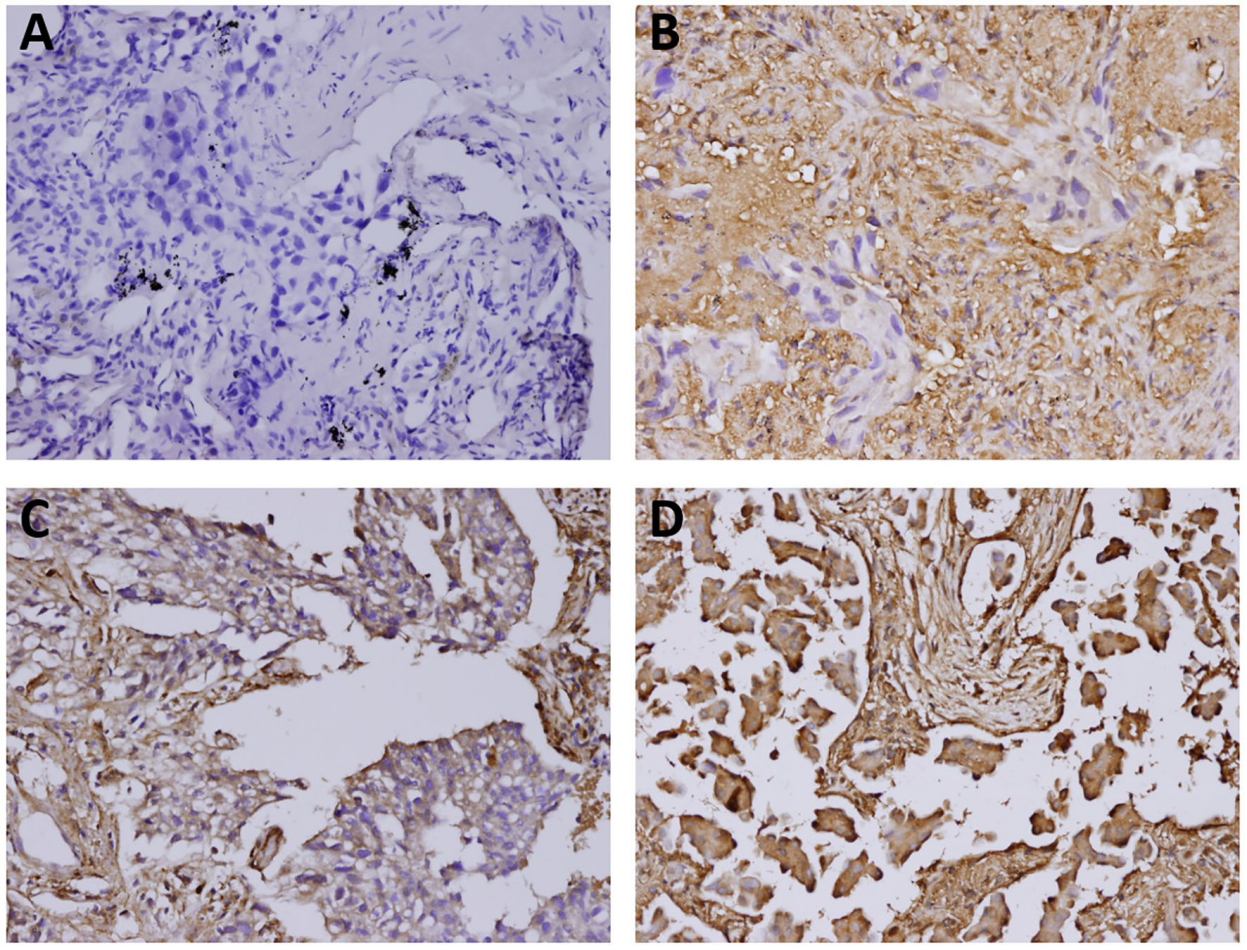
The immunostaining features of tumor galectin-1 expression. The percentages of positive staining for galectin-1 in the tumor part were scored as follows: **(A)** Score 0 for <10% positive staining; **(B)** Score 1 for 10% to <40% positive staining; **(C)** Score 2 for 40% to <70% positive staining; **(D)** Score 3 for at least 70% positive staining. Original magnification 200×.

**Figure 2:**
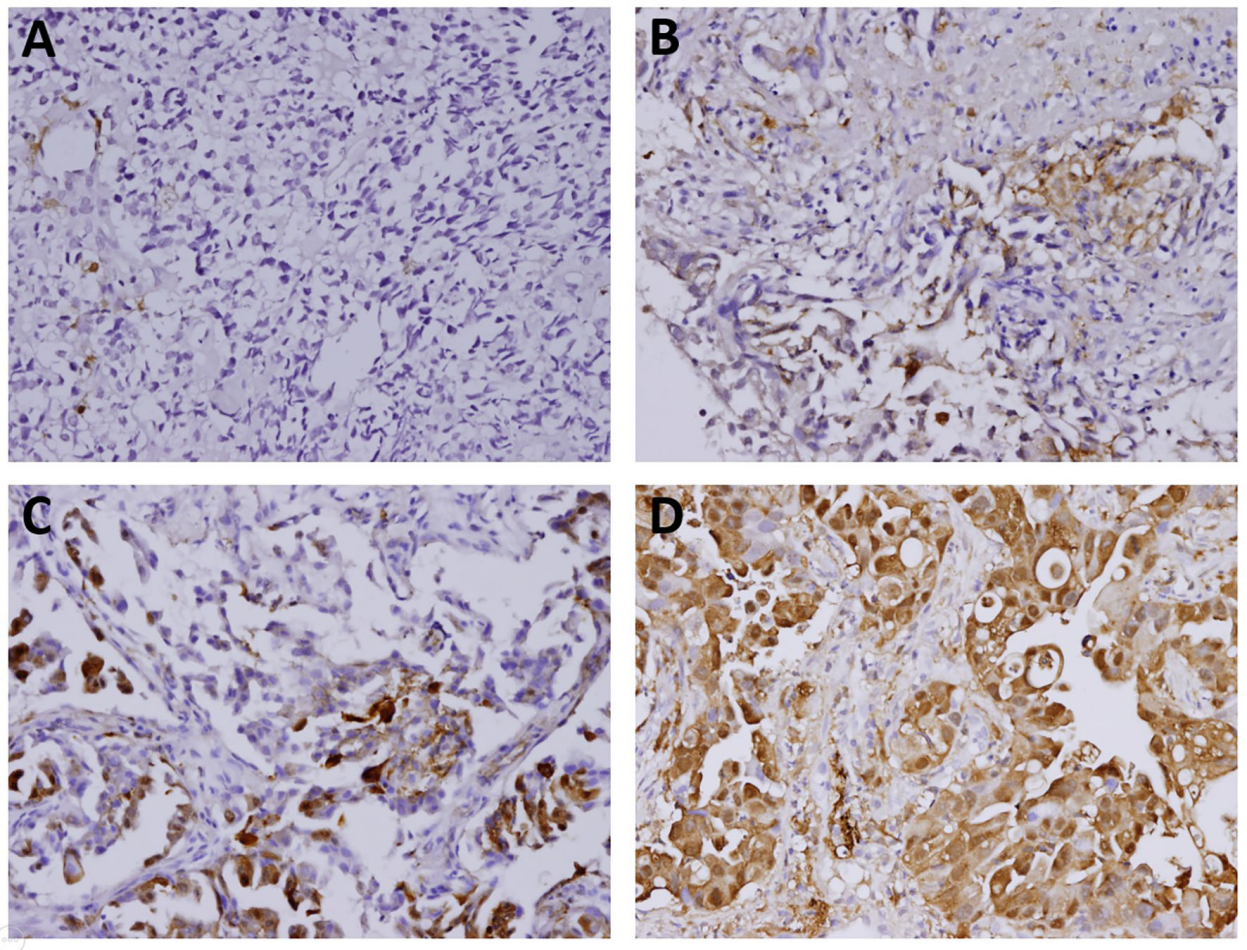
The immunostaining features of tumor galectin-3 expression. The percentages of positive staining for galectin-3 in the tumor part were scored as follows: **(A)** Score 0 for <10% positive staining; **(B)** Score 1 for 10% to <40% positive staining; **(C)** Score 2 for 40% to <70% positive staining; **(D)** Score 3 for at least 70% positive staining. Original magnification 200×.

The immunostaining features of galectin-1 and galectin-3 in the tumor cells and surrounding stroma were evaluated by a single pathologist who were blinded to the clinical treatment outcomes. The percentages of positive staining for galectin-1 and galectin-3 in the tumor part and stroma part were scored as follows: 0 for <10% positive staining; 1 for 10% to <40% positive staining; 2 for 40% to <70% positive staining; 3 for at least 70% positive staining (**Figures 1** and **2**). Both score 2 and score 3 were designated as overexpression of galectin-1 or galectin-3 except that score 1 of galectin-3 in stroma was also regarded as overexpression due to lack of score 2 or score 3 of galectin-3 expression in stroma.

### Statistical Analysis

The clinical treatment outcomes, including overall (OS), locoregional progression-free (LRPFS), and distant metastasis-free (DMFS) survivals were calculated using the Kaplan–Meier method. OS was calculated from the date of initial diagnosis, and both LRPFS and DMFS were calculated from the date of starting radiation therapy. Univariable and multivariable Cox regression analyses were used to check possible prognostic factors of OS, LRPFS, and DMFS. The factors, including gender, age, T stage, N stage, tumor galectin-1, stroma galectin-1, tumor galectin-3, stroma galectin-3, radiation dose, and neoadjuvant chemotherapy, were evaluated in both univariable and multivariable analyses. The results for prognostic factors were presented as hazard ratio (HR) with 95% confidence interval (CI). The *p*-values were two-sided and less than 0.05 was considered to be statistically significant. The statistical analyses were calculated by SPSS Statistics 22.0 (IBM Corp., Armonk, NY).

## Results

The characteristics of patients are listed in **Table 1**. Most patients were diagnosed as locally advanced stage (T3−T4 stage: 78% and N2−N3 stage: 68.3%). Overexpression of tumor galectin-1 and galectin-3 were found in 26.8% and 19.5% of patients, respectively. Overexpression of stroma galectin-1 and galectin-3 was found in 85.4% and 39.0% of patients, respectively.

**Table 1:** Patient characteristics (*N* = 41).

Parameters	All	Tumor Galectin-1	Tumor Galectin-3
	*N* = 41, *N* (%)	Score 2−3, *N* = 11, *N* (%)	Score 2−3, *N* = 8, *N* (%)
Gender			
Female	18 (43.9)	5 (45.5)	2 (25.0)
Male	23 (56.1)	6 (54.5)	6 (75.0)
Age (years)			
Median	62.9	56.9	66.1
Range	39.9−81.2	45.9−70.2	45.9−78.8
<65	22 (53.7)	7 (63.6)	4 (50.0)
≥65	19 (46.3)	4 (36.4)	4 (50.0)
T3−T4			
No	9 (22.0)	4 (36.4)	2 (25.0)
Yes	32 (78.0)	7 (63.6)	6 (75.0)
N2−N3			
No	13 (31.7)	2 (18.2)	2 (25.0)
Yes	28 (68.3)	9 (81.8)	6 (75.0)
Tumor Galectin-1			
Score 0−1	30 (73.2)	−	3 (37.5)
Score 2−3	11 (26.8)	−	5 (62.5)
Stroma Galectin-1			
Score 0−1	6 (14.6)	1 (9.1)	1 (12.5)
Score 2−3	35 (85.4)	10 (90.9)	7 (87.5)
Tumor Galectin-3			
Score 0−1	33 (80.5)	6 (54.5)	−
Score 2−3	8 (19.5)	5 (45.5)	−
Stroma Galectin-3*			
Score 0	25 (61.0)	2 (18.2)	2 (25.0)
Score 1	16 (39.0)	9 (81.8)	6 (75.0)
RT dose (Gy)			
<60	12 (29.3)	1 (9.1)	2 (25.0)
≥60	29 (70.7)	10 (90.9)	8 (75.0)
Neoadjuvant Chemotherapy			
No	34 (82.9)	8 (72.7)	6 (75.0)
Yes	7 (17.1)	3 (27.3)	2 (25.0)

RT, radiation therapy

*No score 2−3 of galectin-3 expression in stroma.

**Figure 3:**
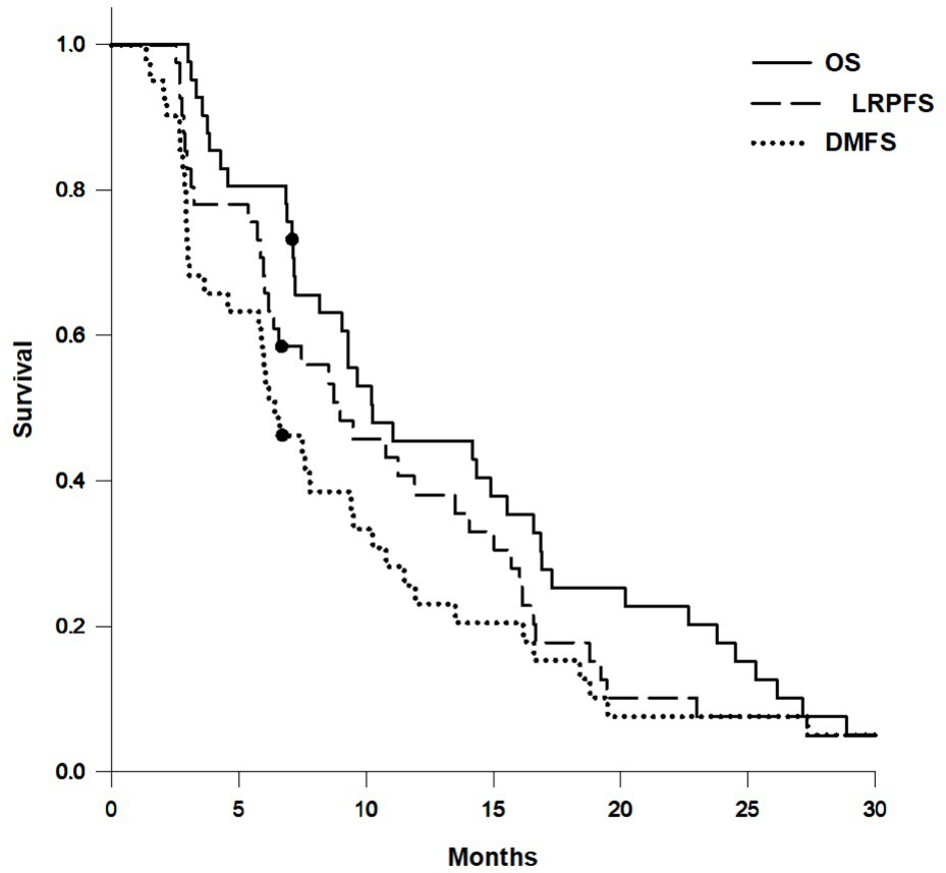
Survival curves of overall survival (OS), locoregional progression-free survival (LRPFS), and distant metastasis-free survival (DMFS) for the whole population.

The median follow-up time was 10.2 months (range, 3.0–204.7 months; mean, 20.0 months). Nearly all patients were followed up until death, except for one patient due to loss of follow-up after 7.1 months. There were 3 patients with first recurrence at locoregional area and 14 patients with first recurrence at distant site. The median OS, LRPFS, and DMFS were 10.3 months, 9.0 months, and 6.4 months, respectively. The actuarial 1-year OS, LRPFS, and DMFS rates were 45.4%, 38.2%, and 23.2%, respectively. The actuarial 2-year OS, LRPFS, and DMFS rates were 17.7%, 7.6%, and 7.7%, respectively. The survival curves of OS, LRPFS, and DMFS for the whole population using the Kaplan–Meier method are presented as **Figure 3**.

**Figure 4:**
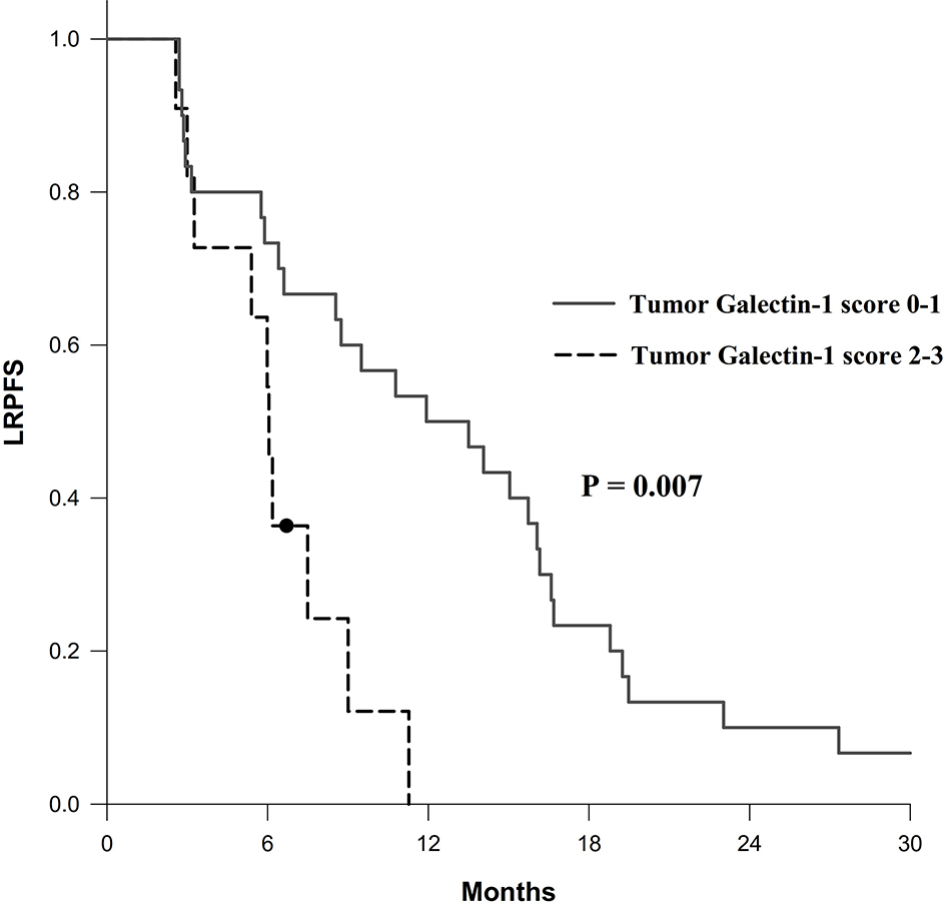
Survival curve of locoregional progression-free survival (LRPFS) according to overexpression (score 2–3) versus non-overexpression (score 0–1) of tumor galectin-1. The *p*-value was calculated using log-rank test.

The results of univariable and multivariable Cox regression analyses of possible prognostic factors for OS, LRPFS, and DMFS rates are listed in **Tables 2**–**4**, respectively. Overexpression of tumor galectin-1 was the most significant prognosticator to predict worse LRPFS in both univariable (hazard ratio [HR] = 3.18; *p* = 0.007) and multivariable analyses (HR = 3.49; *p* = 0.022). The survival curve of LRPFS using the Kaplan–Meier method for overexpression versus non-overexpression of tumor galectin-1 is presented as **Figure 4**.

**Table 2:** Univariable and multivariable Cox regression analyses of OS.

Variables	Univariable analysis			Multivariable analysis		
	HR	95% CI	*p*	HR	95% CI	*p*
Male	1.95	1.01–3.76	0.048	1.89	0.76−4.69	0.171
Age ≥ 65 years	1.69	0.90–3.19	0.105	1.47	0.72−3.01	0.294
T3−T4	0.86	0.40–1.83	0.690	0.73	0.29−1.82	0.496
N2−N3	1.37	0.67–2.82	0.388	0.97	0.37−2.53	0.953
Tumor Galectin-1	1.75	0.83–3.68	0.142	2.80	0.93−8.40	0.066
Stroma Galectin-1	2.02	0.78–5.26	0.148	1.51	0.44−5.19	0.516
Tumor Galectin-3	1.24	0.56–2.74	0.595	0.59	0.20−1.69	0.326
Stroma Galectin-3	1.26	0.66–2.41	0.491	0.95	0.36−2.47	0.912
RT dose ≥ 60 Gy	0.69	0.34–1.41	0.312	0.56	0.24−1.32	0.184
Neoadjuvant Chemotherapy	0.65	0.28–1.49	0.307	0.65	0.24−1.73	0.386

OS, overall survival; RT, radiation therapy; HR, hazard ratio; CI, confidence interval. The p-values were calculated using log-rank test.

**Table 3:** Univariable and multivariable Cox regression analyses of LRPFS.

Variables	Univariable analysis			Multivariable analysis		
	HR	95% CI	*p*	HR	95% CI	*p*
Male	1.95	1.01–3.77	0.047	2.26	0.85−6.01	0.101
Age ≥ 65 years	1.80	0.94–3.43	0.076	1.43	0.69−2.97	0.344
T3−T4	0.81	0.38–1.72	0.581	0.75	0.30−1.92	0.553
N2−N3	1.59	0.78–3.25	0.205	1.09	0.42−2.81	0.860
Tumor Galectin-1	3.18	1.37–7.41	0.007	3.49	1.20−10.14	0.022
Stroma Galectin-1	2.20	0.85–5.70	0.106	1.47	0.44−4.96	0.531
Tumor Galectin-3	1.71	0.78–3.76	0.184	0.69	0.25−1.90	0.472
Stroma Galectin-3	1.48	0.77–2.85	0.244	1.27	0.50−3.22	0.618
RT dose ≥ 60 Gy	0.75	0.37–1.51	0.423	0.63	0.27−1.47	0.281
Neoadjuvant Chemotherapy	0.95	0.42–2.17	0.906	1.13	0.41−3.07	0.814

LRPFS, locoregional progression-free survival; RT, radiation therapy; HR, hazard ratio; CI, confidence interval. The p-values were calculated using log-rank test.

**Table 4:** Univariable and multivariable Cox regression analyses of DMFS.

Variables	Univariable analysis			Multivariable analysis		
	HR	95% CI	*p*	HR	95% CI	*p*
Male	1.25	0.65–2.41	0.501	1.69	0.70−4.07	0.242
Age ≥ 65 years	1.37	0.73–2.59	0.325	1.34	0.64−2.78	0.440
T3−T4	0.88	0.41–1.87	0.739	1.17	0.46−2.97	0.738
N2−N3	1.54	0.76–3.12	0.234	1.48	0.56−3.91	0.434
Tumor Galectin-1	2.43	1.06–5.54	0.035	1.76	0.62−4.98	0.290
Stroma Galectin-1	1.68	0.65–4.35	0.282	1.03	0.31−3.49	0.957
Tumor Galectin-3	1.22	0.55–2.68	0.625	0.50	0.18−1.44	0.201
Stroma Galectin-3	2.02	1.03–3.96	0.040	2.22	0.90−5.47	0.084
RT dose ≥ 60 Gy	1.13	0.56–2.27	0.730	1.19	0.51−2.76	0.687
Neoadjuvant Chemotherapy	1.52	0.66–3.48	0.327	2.47	0.87−7.04	0.090

DMFS, distant metastasis-free survival; RT, radiation therapy; HR, hazard ratio; CI, confidence interval. The p-values were calculated using log-rank test.

**Figure 5:**
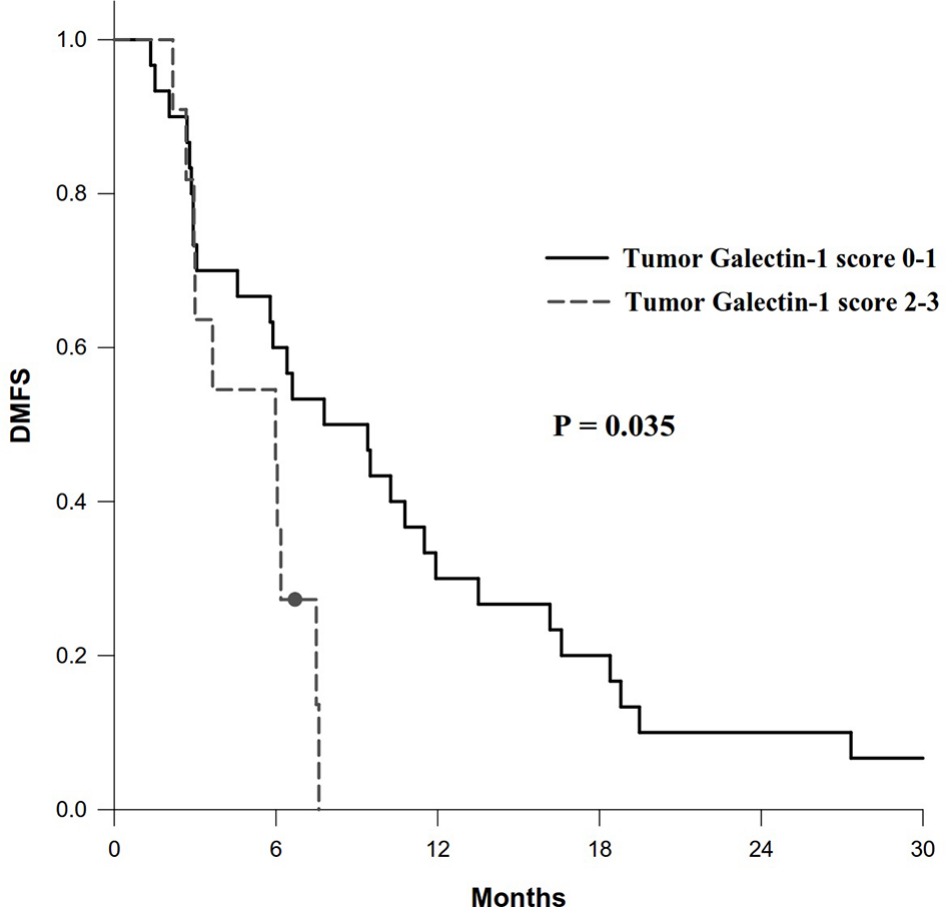
Survival curve of distant metastasis-free survival (DMFS) according to overexpression (score 2–3) versus non-overexpression (score 0–1) of tumor galectin-1. The *p*-value was calculated using log-rank test.

In addition, patients with overexpression of tumor galectin-1 had the trend of worse OS (HR = 2.80; *p* = 0.066) in multivariable analysis, and significantly worse DMFS (HR = 2.43; *p* = 0.035) in univariable analysis. The survival curve of DMFS using the Kaplan–Meier method for overexpression versus non-overexpression of tumor galectin-1 is presented as **Figure 5**.

Other significant prognostic factors comprised male patients for worse OS (*p* = 0.048) and LRPFS (*p* = 0.047) in univariable analyses and overexpression of stroma galectin-3 for worse DMFS in univariable analysis (*p* = 0.040). Overexpression of tumor galectin-3 did not have any significant effect on survival outcomes in this cohort.

## Discussion

In this study, we found that overexpression of tumor galectin-1 was the most significant prognosticator to predict worse LRPFS in patients with lung adenocarcinoma after thoracic radiation therapy without concurrent chemotherapy. Since radiation therapy was the main locoregional therapy modality in this population, the result implied that tumor galectin-1 might be a biomarker to predict radiation response in the patients with lung adenocarcinoma.

Several studies had proposed the role of tumor galectin-1 in the clinical outcomes of non-small cell lung cancer. Carlini et al. (18) reported that high galectin-1 expression had a poorer OS in a cohort of 103 non-small cell lung cancer patients. Schulkens et al. (19) identified that galectin-1 was a significant prognostic factor for OS and progression-free survival (PFS) by performing extensive galectin expression profiling in a cohort of 87 non-small cell lung cancer patients. Zheng et al. (20) presented that positive expression of tumor galectin-1 could predict worse OS and PFS in a cohort of 96 surgically resected lung adenocarcinoma patients. In our study, we also found that overexpression of tumor galectin-1 had the trend of worse OS, and significantly worse LRPFS and DMFS in a cohort of 41 lung adenocarcinoma patients after radiation therapy. This study firstly reported the association of tumor galetin-1 in the clinical outcomes of lung adenocarcinoma after radiation therapy.

Several investigations had explored the possible mechanism of galectin-1 promoting tumor progression and chemoresistance in lung adenocarcinoma. Wu et al. (21) proposed that galectin-1 was involved in tumor invasion and metastasis by increasing matrix metalloproteinase (MMP)-9 and MMP-2 expression and reorganizing actin cytoskeletons. Chung et al. (9) discovered that galectin-1 promoted lung adenocarcinoma progression and chemoresistance by upregulating p38 MAPK, ERK, and cyclooxygenase-2 (COX-2). Zhou et al. (10) reported that galectin-1 overexpressed in CD133-positive lung adenocarcinoma cells, featuring higher invasive and metastatic abilities, and played a major role in tumorigenesis and invasiveness *via* COX-2/PGE2 and AKT/mTOR pathways. Zhang et al. (22) found that galectin-1 gene silencing would improve the sensitivity of lung adenocarcinoma cells to cisplatin *in vivo* and *in vitro*. However, there was a lack of studies to explore the possible mechanism of radioresistance in lung adenocarcinoma, which prompted us to go into study.

As for galectin-3, previous studies had showed the inconsistent association of galectin-3 in the clinical outcomes of non-small cell lung cancer. Mathieu et al. (23) reported that nuclear galectin-3 expression is a predictive factor of recurrence in a cohort of 286 surgically resected lung adenocarcinoma and squamous cell carcinoma patients. Kosacka et al. (24) revealed no prognostic value of galectin-3 expression in non-small cell lung cancer. Kataoka et al. (25) presented that galectin-3 expression in tumor cells could serve as a predictive factor for recurrence (mainly distal recurrence) in patients with non-small cell lung cancer. Kao et al. reported that low galectin-3 expression is associated with metastasis in surgically resected T1 lung adenocarcinoma. In our study, we found that overexpression of stroma galectin-3 would predict worse DMFS in univariable analysis; however, overexpression of tumor galectin-3 had no significant effect on the survival outcomes in the patients with lung adenocarcinoma after radiation therapy.

The tumor stroma could change during malignancy and might have an influence on tumor progression (26). We were curious about the percentage of overexpression of galectin-1 and galectin-3 and their prognostic impact on survival outcomes. In this study, we found higher overexpression of galectin-1 (85.4%) than galectin-3 (39.0%) in the stroma part; however, there was no prognostic impact of stroma galectin-1. Overexpression of stroma galectin-3 could predict worse DMFS in univariable analysis but not in multivariable analysis.

The patient being male was the other significant prognostic factor for worse OS and LRPFS in univariable analyses in our study. Previous literature also showed the same finding. Sakurai et al. (27) reported that male patients had significant worse OS and disease-specific survival in a large cohort of 8,168 lung adenocarcinoma patients. Watanabe et al. (28) revealed that male patients had obviously shorter disease-free interval in lung adenocarcinoma. Kawaguchi et al. (29) presented that male patients had worse OS in the never-smokers with non-small cell lung cancer mainly comprising adenocarcinoma.

The survival outcomes in our study from patients undergoing conventional 2D radiotherapy (2DRT) in the old non-concurrent chemotherapy era were much poorer than those in the report from patients in the concurrent chemotherapy era. The 2-year OS rate in our study was only 17.7%, which was only one-half of 35.6% reported from a meta-analysis (30) including patients with concurrent chemotherapy. With application of much more conformal and precise radiation therapy techniques in the modern era, the reported survival outcomes in the patients with locally advanced non-small cell lung cancer were much better than those reported in the conventional 2DRT era. In a well-known clinical trial (31) of concurrent chemoradiotherapy with or without adjuvant durvalumab immunotherapy, the 2-year OS rate could be as high as 55.6% in the placebo arm and 66.3% in the durvalumab arm.

The main limitations of this study are its limited sample size and the study being retrospective in nature, which may include inherent selection bias. In addition, the enrolled patients were in the old era, and some parts of the charts might be destroyed. The patient characteristics including clinical (such as performance status) or pathological variables (such as tumor grade) are not available, which may bias our results. Finally, the result of treatment outcome is limited to patients without concurrent chemotherapy, which cannot be presented as the treatment outcome of current standard therapy (concurrent chemoradiotherapy with adjuvant durvalumab immunotherapy).

## Conclusion

We report the treatment outcome of patients with lung adenocarcinoma after thoracic radiation therapy without concurrent chemotherapy. The overexpression of tumor galectin-1 is the most significant prognosticator for poor LRPFS. Future research on the mechanism of galectin-1 affecting radiation response in lung adenocarcinoma may be worth exploring.

## Data Availability Statement

The datasets generated for this study are available on request to the corresponding authors.

## Ethics Statement

The studies involving human participants were reviewed and approved by Chang Gung Medical Foundation Institutional Review Board. The ethics committee waived the requirement of written informed consent for participation.

## Author Contributions

C-CHu, I-CC, C-CHs, and E-YH were involved in the conception and design. C-CHu, I-CC, Y-LS, H-LL, Y-CC, and J-YC were involved in the preparation, analysis, and interpretation of the data. C-CHu and I-CC drafted the paper. C-CHs and E-YH revised it critically for intellectual content. All authors contributed to the article and approved the submitted version.

## Funding

The work was supported in part by grants from Chang Gung Medical Foundation/Chang Gung Memorial Hospital (CMRPG8H1001 and CMRPG8H1002) to C-CHu.

## Conflict of Interest

The authors declare that the research was conducted in the absence of any commercial or financial relationships that could be construed as a potential conflict of interest.

## Publisher’s Note

All claims expressed in this article are solely those of the authors and do not necessarily represent those of their affiliated organizations, or those of the publisher, the editors and the reviewers. Any product that may be evaluated in this article, or claim that may be made by its manufacturer, is not guaranteed or endorsed by the publisher.
